# Selective prevention programs for children from substance-affected families: a comprehensive systematic review

**DOI:** 10.1186/1747-597X-7-23

**Published:** 2012-06-12

**Authors:** Sonja Bröning, Karol Kumpfer, Katja Kruse, Peter-Michael Sack, Ines Schaunig-Busch, Sylvia Ruths, Diana Moesgen, Ellen Pflug, Michael Klein, Rainer Thomasius

**Affiliations:** 1 Center for Psychosocial Medicine; German Center for Addiction Research in Childhood and Adolescence, University Medical Center Hamburg-Eppendorf, Martinistraße 52 D-20246, Hamburg, Germany; 2 College of Health; Department of Health Promotion and Education, University of Utah, 1901 E South Campus Dr. Rm 2142, Salt Lake City 84112, UT, USA; 3 German Institute for Addiction and Prevention Research, Catholic University of Applied Sciences Nordrhein-Westfalen, Wörthstraße 10, D-50668, Köln, Germany

**Keywords:** Children of alcoholics, Children of substance abusers, Prevention programs, Familial substance use

## Abstract

Children from substance-affected families show an elevated risk for developing own substance-related or other mental disorders. Therefore, they are an important target group for preventive efforts. So far, such programs for children of substance-involved parents have not been reviewed together. We conducted a comprehensive systematic review to identify and summarize evaluations of selective preventive interventions in childhood and adolescence targeted at this specific group. From the overall search result of 375 articles, 339 were excluded, 36 full texts were reviewed. From these, nine eligible programs documented in 13 studies were identified comprising four school-based interventions (study 1–6), one community-based intervention (study 7–8), and four family-based interventions (study 9–13). Studies’ levels of evidence were rated in accordance with the Scottish Intercollegiate Guidelines Network (SIGN) methodology, and their quality was ranked according to a score adapted from the area of meta-analytic family therapy research and consisting of 15 study design quality criteria. Studies varied in program format, structure, content, and participants. They also varied in outcome measures, results, and study design quality. We found seven RCT’s, two well designed controlled or quasi-experimental studies, three well-designed descriptive studies, and one qualitative study. There was preliminary evidence for the effectiveness of the programs, especially when their duration was longer than ten weeks and when they involved children’s, parenting, and family skills training components. Outcomes proximal to the intervention, such as program-related knowledge, coping-skills, and family relations, showed better results than more distal outcomes such as self-worth and substance use initiation, the latter due to the comparably young age of participants and sparse longitudinal data. However, because of the small overall number of studies found, all conclusions must remain tentative. More evaluations are needed and their quality must be improved. New research should focus on the differential impact of program components and delivery mechanisms. It should also explore long-term effects on children substance use, delinquency, mental health, physical health and school performance. To broaden the field, new approaches to prevention should be tested in diverse cultural and contextual settings.

## Background

Substance misuse and dependency severely impact physical and mental health. They are often accompanied by comorbid mental disorders and behavioral problems, especially when consumption begins early in life [1,2]. Increasing rates of adolescents’ legal and illicit substance use across industrialized countries in recent years [3], as well as risky consumption patterns in European and American youth [4-6], have elevated community concerns. Diverse prevention efforts have resulted from these concerns under which universal prevention approaches still remain the most common. By intervening early in life, these efforts aim at turning developmental pathways away from substance use problems and the danger of their developing into substance use disorders (SUDs) [7]. There is considerable research evidence for the effectiveness of substance abuse prevention programs. They appear to be strongest when they draw on social influence concepts, when they target high-risk groups early, for example from disadvantaged community areas [8], and when they are family-focused [9-11]. Effect sizes are small for universal youth-only substance abuse prevention programs. For selective and indicated family-focused prevention interventions, up to nine times greater effect sizes have been documented [12-17]. To increase the impact of drug prevention, universal prevention needs to be supplemented by more specific prevention programs geared to the needs of different populations at risk for developing a problematic use of substances.

The negative impact of parental drug use has been documented by a multitude of studies and reviews, especially for children of alcoholics. It includes physical, psychological, and cognitive consequences for children’s development [18-20]. Children and adolescents affected by parental drug use show higher rates of externalizing and internalizing problems such as antisocial behavior, emotional problems, attention deficits, or social isolation [21]. With regard to substance use problems, their records more often show an early onset of substance consumption [22,23], earlier drunkenness experiences [24], increased binge drinking rates [25], and an elevated risk for developing substance use disorders at a younger age than comparable peers [26]. Approximately 33 to 40 percent of all children with a substance using parent will develop a substance use disorder themselves [27,28].

Substance problems are transmitted to the next generation via several pathways, especially genetic disposition [29,30], behavioral, and cognitive processes [31,32]. Family environmental characteristics such as problematic family relationships [21], family conflict, or absence of supportive parenting [33] play an important part in transmitting substance use problems to offspring. The same goes for positive expectations about the effects of substance consumption acquired in familial context [34,35]. Learning from the model substance-dependent parents set, children learn to use substances as coping strategies in stressful and difficult times [36-38]. Family environment can also be a significant resource. Family attachment or bonding, monitoring, and communication of positive family values and expectations are strong protective factors in preventing substance use and abuse [9]. Recent epigenetic animal research on the role of the prenatal and postnatal environment on expression of inherited diseases such as substance use disorders suggests that one of the most protective factors is nurturing parenting [39-41].

These findings have led practitioners and researchers to target children of substance using parents with family-focused prevention interventions that increase supportive and nurturing parenting. These family interventions are expected to reduce the risk for later substance abuse, and, consequently, the high societal costs of delinquency, mental and physical disorders, and child maltreatment [9-11]. Considering the research mentioned above, they seem more promising than interventions targeting only parents or only children. However, in the case of substance-affected families, parents frequently are not willing to participate in such a program. Nevertheless, they may endorse the benefits their children receive from a prevention program. Resilience theory and research [42,43] demonstrate that children’s development is influenced by their own cognitive appraisal of a life with a substance-abusing parent as well as by their emotional and behavioral strategies of coping with difficult situations arising from parental substance use. Consequently, interventions enhancing these skills in children seem a further promising prevention form. They mainly focus on children aged 8–12 years since these children are old enough for cognitive teaching strategies while not yet in puberty where own substance consumption problems commence. Child-focused programs are frequently delivered in a peer-group format, for instance in a school setting, [17,44,45] so children can benefit from positive peer influence and mutual support.

While there is considerable evidence for the effectiveness of universal prevention programs [46-48], the field is only just evolving in relation to selective prevention programs for children of substance abusers. To date, programs specifically geared to this high-risk population have not yet been reviewed together, even though some of them have been mentioned in more general systematic reviews on the (universal) prevention of tobacco, alcohol, and drugs [12,14,49,50]. One article reviews prevention for children of parents who abuse only alcohol [8], while another targets children of illicit drug users [51]. Woolfall and Sumnall [52] as well as Kumpfer [53] focus on outcome measures in evaluating interventions for children of substance using parents, but do not analyze the interventions. Barnard and McKeganey [54] review key evaluated interventions for families with SUD problems and conclude that interventions focusing directly on children’s needs are important but scarce. The aim of this paper is to gather the evidence on prevention programs designed specifically for *children* with a substance using parent.

We choose a systematic review approach because a meta-analytic strategy does not seem appropriate considering the small body of research and its heterogeneous designs. We focus on child outcomes, such as child functioning or child substance use, and on family attributes that, by definition, include child outcomes such as family cohesion. We also compare the interventions described in the studies with regard to similarities and differences and review the design quality of the studies. In the following, we describe our approach and methods. Then we present an overview of the relevant programs, their contents and their evaluation. The corresponding studies are rated according to their evidence level and design quality. In the subsequent discussion we integrate the results of the review based on the aspects mentioned above and draw conclusions for research and practice.

## Methods

### Identification of studies and inclusion criteria

 Our methodology was guided by guidelines for current systematic literature reviews [55,56]. All studies describing or investigating validly established effects of preventive interventions on children and adolescents with substance abusing parents (or on affected families as a whole entity) were included in the review. We searched for relevant studies published during the period of 1994–2009, thus choosing a time span of 15 years. This is broad enough to incorporate a larger number of studies than the more common 10-year-span, but not too broad to ensure the comparability of the scientific methodology used in the studies. Using the search terms “prevention AND child* AND (parents AND (addict* OR alcohol))” delivered the most results. We used this combination to search the following databases: Cochrane database of systematic reviews, Ovid, MEDLINE(R), EMBASE, PsycINFO and PSYNDEXplus. The age of the target population ranged from 0–17 years, and only English or German literature was included. After removing duplicates our initial search on January 2, 2010, generated 348 articles. Of these, nine articles were identified as potentially relevant, and full texts were obtained. 339 articles were excluded because they did not deal with prevention programs specifically for children of substance using parents or affected families. Their topics can be summarized as follows: addiction and violence (12 articles), programs for substance using parents (5 articles), effects of parental addiction on children (37 articles), predicting SUD development (58 articles), addiction prevention in general (84 articles), alcohol in general (24 articles), other topics (119 articles). In addition to the search described above, an extensive hand search was conducted in public search engines (using keywords, program and researcher names) and by screening the references of the full texts. This yielded additional 27 articles, resulting in a total number of 36 potential articles to be included. The articles were examined by two independent reviewers (Katja Kruse and Gurli Herrmann) according to the following inclusion criteria:

#### Evidence-based outcome

All studies describing or investigating validly established effects of prevention programs on the target group considered here were included. Thus, a text was excluded if only program characteristics are described, but no empirical evidence is reported, or if case histories, case studies, or a single-case study are used as evidence for the effectiveness of the program.

#### Publication characteristics

The study is published in a peer-reviewed national or international scientific journal.

#### Age of child / children

The program is targeted at children and/or adolescents aged 0–17 years or at families with children of this age.

#### Substance consumption of parent(s)

At least one parent or legal guardian uses alcohol and/or other psychotropic substances (legal or illegal) in a risky or problematic way, or is dependent on at least one substance. Therefore, an article was excluded if the described program belongs to the category of universal prevention programs (e.g., whole school-class), or selective prevention programs for a more unspecific risk-group (e.g., whole school-class from inner-city setting). Regarding parental consumption, a DSM- or ICD-diagnosis was not required so as not to further diminish the few relevant studies identified. Thus, parental consumption was operationalized differently in the various studies and could be assessed via self-report (questionnaire, interview) of children, parents, or by report of third parties, such as teachers, therapists, or medical practitioners.

#### Type of intervention

The intervention is preventive and targets children and/or adolescents from substance-using homes. Thus, articles were excluded when programs described therein were classified as indicated prevention programs (e.g., when children and/or adolescents were already showing harmful substance use) or as therapeutic interventions (e.g., for children with mental problems diagnosed according to child and adolescent psychiatric standards; for information on different prevention forms see [49]).

**Figure 1:**
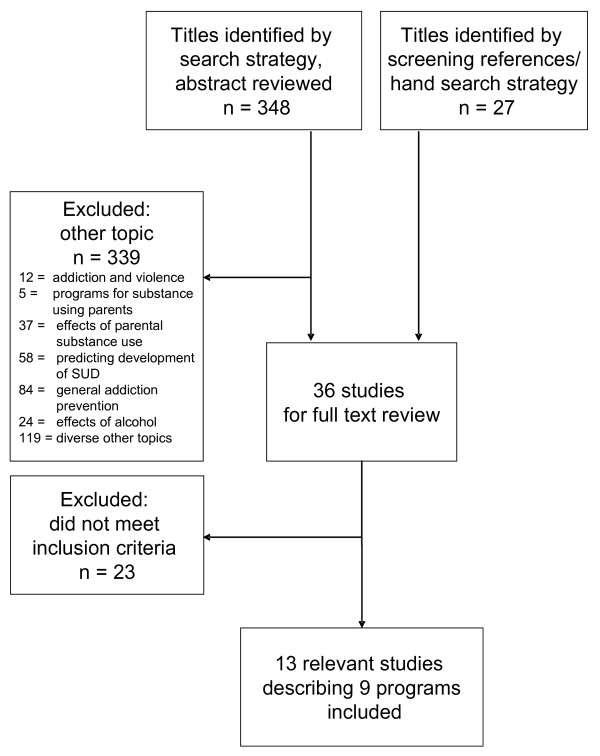
Study selection process.

Differences between reviewers were resolved by consensus. They occurred when studies were ambiguous about inclusion criteria (e.g., age-group of the children) or when their methodology seemed disputable. When there was doubt, and in view of the small number of relevant texts, the study was included. At the end of the review process 13 studies evaluating nine programs met our inclusion criteria (see Figure 1 for an overview of the selection process).

### Quality criteria

The studies’ levels of evidence were rated according to the methodology for grading recommendations in evidence-based guidelines of the *Scottish Intercollegiate Guidelines Network* (SIGN), ranging from Ia to IV [57,58] see Table 1. This system is internationally accepted and used by guideline developers. The *Society for Prevention Research*[59] as well as Kumpfer and Alvarado [47] have published similar rating scales that include a even higher level of evidence of effectiveness specifying evidence from multiple randomized, controlled trials (RCTs) that include independent research teams and criteria for the quality of the research. In our case, however, the older version of the SIGN classification system was used, as it seemed better suited to distinguish between studies with comparably low overall quality levels. It contains four levels of evidence that are further differentiated into sub-levels, as shown in Table 1.

**Table 1:** Levels of evidence according to the Scottish Intercollegiate Guidelines Network (SIGN, 1999)

**Levels of evidence**	
Ia	Evidence from a systematic review or meta-analysis of randomized controlled trials (RCT’s)
Ib	Evidence from at least one RCT
IIa	Evidence from at least one well-designed controlled study without randomization
IIb	Evidence from at least one well-designed quasi-experimental study, such as a cohort study
III	Evidence from a well-designed non-experimental descriptive studies, such as comparative studies, correlation studies, case–control studies and case series
IV	Expert committee reports, opinions and/or clinical experience of respected authorities

Since only a small number of adequate studies matched our inclusion criteria we included not only RCTs but also studies with lower levels of evidence. To quantify the quality of the included studies more precisely within the SIGN categories and to enable a ranking list of the studies, a methodology quality score (MQS) was applied that we adapted to our subject matter from a score used in the area of meta-analytic family therapy research [60,61]. With this score we examined and rated studies according to 15 criteria of study design quality (see Table 2). The total score rates the design quality of a study as “low” (score 0–14), “modest” (score 14.5-19), “good” (score 19.5-24) or “very good” (score 24.5-30).

**Table 2:** Methodology Quality Score (MQS; adapted from Gurman and Kniskern 1978; Stanton and Shadish 1997)

	**Criteria and their scores**	**Maximum score**
1	Controlled assignment to treatment conditions (random assignment, matching of total groups or controlled design, without randomization (2.5) matching in pairs)	5
2	Pre-post-measurement of change	5
3	Sample size N = 50-100 (0.5) N = 100-150 (1) N > 150 (1.5)	1.5
4	No contamination of major independent variables: Most important independent variable is valid, i.e., parental substance use proven (2), very likely (1), program implemented by professional staff (3)	5
5	Appropriate statistical analyses	1
6	Data collected via self-assessment *and* expert interviews	1
7	Follow-up in months 1–3 (0.5), 4–6 (1), 7–12 (1.5) 13–18 (2), 19–24 (2.5) >24 (3)	3
8	Evidence of treatment adherence: probably (0.5), certainly, as stated by the authors (1), verifiable (1.5)	1.5
9	Multiple change indices	1
10	Multiple vantage points for assessing outcome, multiple criterion measurement	1
11	Quality of instruments reported	1
12	Simultaneous data collection for the control group; Non-simultaneous data collection for the control group (0.5)	1
13	Outcome assessment: Non-responder – improvement (0.5)Deterioration – improvement (1)	1
14	Therapist-investigator nonequivalence: examiners not involved in program delivery	1
15	Treatment dropouts / intent-to-treat analyses	1

### Effect Sizes

Effect sizes (*ES*) were computed in terms of (unweighted) correlation *r*. Using Fisher’s Z-transformation weighted mean correlations *r*(+) were also computed [62]. This could only be done in studies supplying appropriate data.

## Results

Our search yielded 13 studies [63-75] with nine programs designed specifically for children of substance abusing parents., comprising four school-based interventions (study 1–6), one community-based intervention (study 7–8), and four family-based interventions (study 9–13). Some further studies were consulted for further information on the program or study [76-80]. With the exception of one program conducted in Spain *Family Competence Programme* (FCP)] [63] and another one with partial sampling in Canada [64] (both of these programs being culturally adapted versions of Kumpfer’s *Strengthening Families Program* with Kumpfer as a co-investigator), all programs and their evaluation were conducted in the United States. For an overview on program format, structure, content and participants, see Table 3. For an aggregated overview and effect sizes, see Table 4. For an overview on outcome measures, results and study design quality, see Table 5. In the following, we describe our results using the following approach: first, studies are compared with regard to their design quality. Second, program settings and structure are summarized. Third, study measures, findings, and effect sizes are summarized.

**Table 3:** Program and recruitment characteristics

	**Study**	**Name of intervention**	**Target group and focus of intervention**	**Format**	**Access to participants / recruitment**
**School-based Interventions**					
1	[65]	Stress Management and Alcohol Awareness Program (SMAAP)	School-based group program for 4th-, 5th- and 6th-grade students with problem-drinking parents. Focus: self-esteem, coping behaviors, alcohol expectancies, problem solving, social support. Didactics: theory, practical exercises, homework assignments, complementary “personal trainer component”	8 weekly 90-min sessions	Children identified their parents’ problems after watching a relevant video. Interested children were invited to participate in the program. Parental consent was obtained.
2	[66]	Friends in Need	School-based group program for primary school pupils from drug-involved families. Focus: self-esteem, coping behavior, perception of emotions, group affiliation, „4 C’s” (“you didn’t cause it, you can’t control it; you can’t cure it, you can be okay.”) Didactics: theory, practical exercises, structured sessions, rituals	8 90-min sessions	After a discussion group on feelings about drug use, teachers from three schools that were located in drug-involved neighborhoods identified children whom they believed to be affected by parental drug use. Parental consent was obtained.
3	[68]	School-Based-Support-Groups (SBSG)	School-based group program for students from grades 9 through 12. Focus: knowledge on substance abuse and its impact, family relations, coping strtegies. Didactics: theory, practical exercises, mutual support	14 weekly 60-min sessions	A school-based health center and/or a high school counselor identified students reporting substance use in their family (screening question: “Does anyone in your family drink or take drugs so much that it worries you?”). Parental consent was not obtained.
4	[67]	SBSG	See (3), slightly different format (see format)	15 45-min sessions	The program was introduced by school personnel; interested students were welcome to participate.
5	[69]	Children Having Opportunities in Courage, Esteem and Success (CHOICES)	School-based three-component program for 3rd- and 4th-grade students: 1. “School Support Group”: group meetings. 2. “Healthy Lifestyle Peer Mentors”: ongoing mentoring program for participants; peer mentors received training and attended group meeting. 3. private lessons / homework assistance. Focus: emotions identity and family, coping strategies. Didactics: discussions, videos, practical exercises	11 weekly 60-min sessions, weekly individual 30-min sessions with mentors	A drug related video was shown, children answered two screening questions, children screened positive were interviewed by a student counselor, who assessed program eligibility. Parental consent was obtained.
6	[70]	CHOICES	See (5)	See (5)	See (5)
**Community-based Interventions**					
7	[72]	Teen-Club	Group-program for female teenagers with drug-involved families and a lack of social and family support. Focus: problem solving, health education, social behavior, home visits for crisis intervention. Didactics: theory, motivational leisure activities	Weekly 90-min meetings within two years	Offered by a youth center with a high risk population, no accurate information regarding recruitment provided.
8	[71]	Teen-Club	See (7)	See (7)	See (7)
**Family-based Interventions**					
9	[73]	Focus on Families (FOF)	Family-based program for families with methadone treated parents, sessions with groups of families (partly with children, partly without), combined with home-based case management. Focus: relapse prevention, stabilization and improvement of family management practices. Didactics: motivational elements, discussion, practical exercises, periodical buffer calls for 9 months after program end	32 biweekly 90-min sessions (12 with children) for 16 weeks	Participating families were recruited at two methadone clinics in Seattle.
10	[74]	FOF	See (9)	See (9)	See (9)
11	[64]	Strengthening Families Program (SFP, Utah-Version).	Canadian adaption of the SFP 6–12 Year family based program, developed by Kumpfer & DeMarsh (1983) in 1982, tested with children aged 9 to 12 with at least one parent addicted to alcohol in Ontario and Buffalo, NY. Focus: Strengthening individuals as well as family structures. Didactics: theory, practical exercises, videos, session split into children’s / parent’s groups and joint family sessions	14 weekly 2-3-hour sessions	Recruited from multiple alcohol treatment agencies and community agencies for high-risk families in Ontario and Buffalo.
12	[63]	Family Com-petence Pro-gram (FCP).	Spanish adaptation of the SFP 6–12 Years, see (11). Family-based program for parents and children (aged 6 to 14).	14 weekly 2–3 hour sessions	Interested drug-using parents in the final phase of addiction treatment, and their children.
13	[75]	Safe Haven Program	Adaptation of the SFP (Utah version), see (11) for inner city African-American substance-using families with children aged 6 to 12. Focus: parent training, children’s and family skills training. Didactics: practical exercises, homework, theory	12 weekly sessions	Parents were recruited at a residential drug and alcohol treatment center. Potential participants were interviewed twice to assess their level of interest and potential commitment. Consent of all family members was obtained.

**Table 4:** Study ranking by design quality and key characteristics (Y = yes, N = no, FU = follow-up, min = minutes, f = females, m = males)

**No**	**Program**	**Evidence class**	**Design quality**	**Rank**	**RCT**	**Pre post**	**FU**	**Sample size**	**Age M**	**Setting**	**Dose**	**Key significant findings in favor of children in treatment groups**	**Effect sizes r**
1	SMAAP	Ia	24,5	3	Y	Y	Y	>200	10.1	School	8 x 90 min	knowledge, coping, social behavior	.54 / .24 / .12
2	Friends in Need	Ib	23	5a	Y	Y	N	>200	3-4^th^ grade	School	8 x 90 min	social behavior	qualitative data
3	SBSG	Ib	23	5b	Y	Y	N	100-200	15.5	School	14 x 60 min	knowledge, coping (f)	.37 / .54 (f)
4	SBSG	III	9	11	N	N	N	<50	?	School	15 x 45 min	knowledge, coping, school performance, social behavior	qualitative data
5	CHOICES	Ib	22,5	6	Y	Y	Y	<50	8.8	School	11 x 60 min	self-esteem, school performance	.43 / .52
6	CHOICES	III	10,5	10	N	N	N	50-100	3-4^th^ grade	School	11 x 60 min	social behavior	qualitative data
7	Teen-Club	III	14,5	9	N	N	N	<50	18-22	Youth Center	90 min over 2 years	self-esteem, social behavior	qualitative data
8	Teen-Club	IV	8	12	N	N	N	<50	?	Youth Center	90 min over 2 years	self-esteem, social behavior	qualitative data
9	FOF	Ib	27	2	Y	Y	Y	100-200	10.4	Methadone clinic	32 x 90 min	family functioning	.22
10	FOF	Ib	27,5	1	Y	Y	Y	100-200	22	FU interview	32 x 90 min	lower SUD risk (m), delayed age of onset (m) at FU	OR = 0.80, r = .39
11	SFP	Ib	23,5	4	Y	Y	N	>200	11	Parents in outpatient treatment	14 x 120–180 min	social behavior	.11
12	FCP	IIa	20,5	7	N	Y	N	<50	10.6	Parents in outpatient treatment	14 x 120–180 min	knowledge, social behavior, family functioning	.70 / .44 / .44
13	Safe Haven Program	IIb	19	8	N	Y	N	100-200	7.6	Parents in outpatient treatment	12 x (?) min	externalizing / internalizing symptoms, family functioning	.34 / .29 / .29

**Table 5:** Study quality and characteristics

	**Study**	**Name of intervention**	**Evidence class / design quality**	**Research design**	**Sample**	**Outcome measures**	**Significant results for participants**
**School-based Interventions**							
1	[65]	Stress Management and Alcohol Awareness Program (SMAAP)	Ia / 24,5	Randomized-controlled design, pre-post-tests, 4 points of measurement, 2 wait-list control groups, questionnaire study, self-assessment, and assessment by others (teachers). Analysis: ANCOVA, effect sizes. Limits: recruitment based on self-selection procedures (target group unclear), no consistent intent-to-treat analysis, follow-up only for cohort 1	N = 271 at t_0_ (26% dropout), randomized assignment to three cohorts. characteristics: age M = 10,1 years, 60% female, 50% ethnical minorities	standardized / validated measures	improved program knowledge. improved emotion-focused coping (self-assessment). improved problem-solving ability and social competency (teacher rating). no difference with or without personal trainer component
2	[66]	Friends in need	Ib / 23	Randomized-controlled study design, pre-post-tests, 3 points of measurement, wait-list control group, questionnaire study, self-assessment and assessment by others (teachers and group leaders). Analysis: no information provided. Limits: eligible children identified by school personnel; researchers were involved in delivering the program	N = 206 children (no dropouts reported). assignment to one of 16 groups, 37% female, age: 3^rd^ to 4^th^ grade students, 70% Afro-Americans, 29% Caucasian	standardized / validated measures	pre-test: unusually high levels for loneliness and social isolation compared to norm populations. reduced physical aggression for the intervention group compared to controls. no other significant treatment effects
3	[68]	School-Based-Support- Groups (SBSG)	Ib / 23	Randomized-controlled study design, pre-post-tests, wait-list control group, blinded analysis, questionnaire study, self-assessment and assessment by others (by teachers and group leaders). Analysis: t-test, Chi²-test. Limits: Assignment to groups based on teachers’ assessment, liberal level of significance (0.10), analysis of the relative changes only, no dropout analysis, only self assessments, no effect sizes.	N = 109 at t_0_, (17 % dropout), age: M = 15,5 years, sample. characteristics (post-test): 62% female, 56% ethnical minorities.	standardized / validated measures	improved addiction-related knowledge in the study group, no significant group differences in substance use pre“ valences. improved coping strategies and social integration in the study group (females only). increased medical complaints and diminished social integration in the study group (males only).
4	[67]	SBSG	III / 9	Qualitative design. Analysis: ethnographical methods. Limits: no quantitative data, no objective data collection, very small sample size, self-registration.	N = 21. sample characteristics: 67% female, 33% Latin-American	interviews, records	qualitative findings: improvement of social behavior, school performance, coping strategies and knowledge on program content.
5	[69]	Children Having Opportunities in Courage, Esteem and Success (CHOICES)	Ib / 22,5	Randomized-controlled study design, pre-post-tests, 3 points of measurement, questionnaire study. Analysis: ANOVA, t-test. Limits: very small sample from one school, assignment to group by teachers’ assessment, no self-assessment, only group comparisons.	N = 16, randomized group assignment to one of four groups (group 4 = controls). characteristics: M Age = 8,8 years, 56% female, 81,3% white.	standardized / validated measures	increased self-esteem in the group with combined group program and peer mentor training. increased social skills in the group combining program with peer matching. performance at school: significant values in groups 1 and 3. attitude towards substance use improved significantly in all study groups.
6	[70]	CHOICES	III / 10,5	Questionnaire process evaluation study without standardized scales, self assessment and assessment by others. Analysis: no information given. Limits: no controls, no pre-post-tests, no randomization, recruitment based on teachers’ perceptions, dropouts not considered.	N = 60, 3^rd^ and 4^th^ grade students	per fiat measures	self-assessment: improvement in isolation, loneliness, coping strategies and knowledge on program content. assessment by teachers: improvement in attitudes, school performance, social behavior.
**Community-based Interventions**							
7	[72]	Teen-Club	III / 14,5	Retrospective study, questionnaire and interviews, 1 point of measurement five years after enrollment in the program. Analysis: no information given. Limits: very small sample, no pre-post-tests, no information about recruitment and analysis.	N = 12 Afro-American girls between 18 and 22 years	standardized / validated measures, per fiat measures	study group: went to school for a significantly longer time period, had a better chance of getting a job, fewer depressive symptoms, fewer pregnancies, higher frequency of alcohol consumption (no difference in the amount of alcohol consumption)
8	[71]	Teen-Club	IV / 8	Focus group interview, interpretative analysis. Limits: very small sample, purely qualitative survey without any statistical analysis	N = 11 Afro-American girls	interviews	high program contentedness, decreased risky behavior
**Family-based Interventions**							
9	[73]	Focus on Families (FOF)	Ib / 27	Randomized-controlled study design, pre-post-follow-up tests, 3 points of measurement, interviews, random urine sampling. Analysis: ANOVA, ANCOVA, intent-to-treat analysis. Limits: low representativeness, high selectivity: 25 % of the primarily recruited families refused to participate	N = 130 families, children: N = 177, study group: N =95, control group: N = 82, age M = 10,4 years, 77% of parents white, diagnosed substance use (methadone-clinic)	standardized / validated measures, interviews	hardly any differences between study and control group children, improved family behavior in the study group. significant improvements for the parents in the areas parent skills, drug use, deviant peers, and family management
10	[74]	FOF	Ib / 27,5	Follow up of (9), point of measurement 12 years later, structured interviews. Analysis: based on Cox-Model, intent- to-treat analysis. Limits: Only substance consumption was assessed	sub-sample from (9), N = 151 former FOF- or TAU participants were interviewed, characteristics: age M = 22 years, 57% male, comparison to a general population of similar age.	standardized / validated measures	former participants had significantly higher levels of substance use with a lower age of onset compared to a general population sample. reduced risk of developing substance problems for the study group compared with control group (only males)
11	[64]	Strengthening Families Program (SFP)	Ib / 23,5	Randomized-controlled study design, pre-post-tests. Analysis: ANOVA, Intent- to-Treat-Analysis. Limits: no information about recruitment, only one criterion as dependent variable	N = 280 families. study group = 147, controls = 133. characteristics. children’s age M =11 years, 44% female children	standardized / validated measures (not mentioned in the abstract but in the poster that is referred to)	reduction of Oppositional Defiant Disorder Symptoms in the study group compared to the controls from the parents’ perception
12	[63]	Family Competence Programme (FCP)	IIa / 20,5	Quasi-experimental design, pre-post- tests. Analysis: t-test, ANOVA, effect sizes. Limits: small sample, no randomization, only families from “Proyecto Hombre”, effects were mainly based on self- assessments, no information about undesired results or non-respondents, no information about the distribution of substance amounts, no long-term study, no intent-to-treat analysis.	N = 38 children, study group = 22, controls = 16, characteristics: mean age = 10,6 years, parental drug- dependence was diagnosed	standardized / validated measures	family: significant improvement at post-measurement compared to control group in family involvement, communication and family rules, family satisfaction and organization, relationship between parents and children. parents: parenting behaviors and relationship between parents improved. children: problem behaviors were reduced, social skills and program-related knowledge improved
13	[75]	Safe Haven Program	IIb / 19	Quasi-experimental design, pre-post- tests. Analysis: ANOVA. Limits: no randomization, comparison only between high and low drug use groups, no information on the quality of the used instruments, follow-ups are mentioned but not reported.	N = 88 families with one “ targeted” child each. characteristics: mean age = 7,6 years, 44% female children.	standardized / validated measures	high drug use group: improvement of externalizing / internalizing problem behaviors. low drug use group: fewer school problems. total sample: improved family cohesion, less parental drug use

### Study design quality

Study evidence levels differed in the studies examined here: We found seven RCT’s (Ia/Ib;1/2/3/5/9/10/11), two well designed controlled or quasi-experimential studies (IIa/IIb; 12/13), three well-designed descriptive studies (III; 4/6/7) and one qualitative study (IV; 8). Sample sizes ranged from N = 12 to N = 280. The methodological quality of the studies as rated with the MQS appears quite heterogeneous, with a substantial percentage of very good (1/9/10) or good design quality (2/3/5/11/12), some modest quality designs (7/13), and some studies of low quality (4/6/8). Limitations of the existing body of research are discussed below with regard to future research needs.

### Program settings and structure

Most programs for children from substance-affected homes identified and reviewed in this article were school-based, two were family-based, and one was community-based. Intervention duration and intensity did not vary much. Most programs lasted between 8 and 14 weeks with weekly sessions of approximately 90 minutes. Group sizes were not always reported and usually ranged from 8 to 12 children. Only the community-based intervention (7/8), originally designed for ten weeks, lasted for two years on participants’ request. Program content did vary, but common themes for most of the programs emerged, such as coping with emotions, problem solving, education on drugs and addiction, family relations. Didactics usually included theory and practical exercises, discussion, role-play, and video material in some cases. Some programs pointed out structure and fixed rituals as especially important for the target group at hand. Few programs included several components. One intervention (5/6) included a peer mentoring program and homework assistance (the latter of which was not evaluated). The community-based intervention (7/8) included motivational leisure activities and offered family-based crisis intervention. One family-based program (9/10) combined parent and family sessions with home-based case management. Periodical buffer calls lasted for nine months after the intervention. The family-based programs stemming from the SFP (11/12/13) included a dinner break for participating families, but no further components.

In the school-based programs, parents (substance using parent or his/her spouse) were in no way involved in the program, other than giving their consent for participation of the children. They were also not included in data collection and therefore did not assess children’s behaviors, adjustment or problems, thus creating a possible bias regarding study results. In the community-based program, home-based case management was offered, but it remains unclear as to how extensively it was made use of, so that here too, parental involvement cannot be assumed. Family-based programs differed in the attention given to children, parents, and family sessions. One program for families with a methadone-treated parent (9/10) focused primarily on strengthening the adults in their role as parents and in preventing relapses. There were no sessions solely for the children; about a third of all sessions were conducted as family sessions. The SFP modifications (11/12/13) for families with parents addicted to different substances involved parents and children in joint, but also in separate sessions, parents and children receiving the same amount of time. Hence, individual needs could be accounted for, but the family also was recognized as an entity, and individual developments could be brought together and integrated.

### Study measures and findings

The most frequent outcome measures were knowledge, self-worth, coping, and social behavior. Further attributes such as emotion regulation, depression, health behaviors, substance use, school attachment and performance, family and/or social relationships were also included. All of these outcome measures address parameters known to increase the risk for developing mental disorders [81]. Only two studies considered possible substance use of participating children (7/10). As listed in Table 4, unweighted effect sizes within the studies vary from *r* = .70 for knowledge (study 12) to *r* = .11 for social behavior (study 11). Weighted ES are *r*_*(+)*_ = .55 for knowledge, .34 for coping, .27 for family functioning, and .17 for social behavior (not given in Table 4). Overall, the small amount of eligible studies as well as the heterogeneity of study designs, quality and programs allow nothing more than the following preliminary synthesis of findings:

(a) *Own reduction of substance consumption or abstinence* was evaluated only in some studies, although almost all studies stated this as their ultimate preventive goal. In one study with good design quality (3), no reduction of substance consumption was found for the experimental groups, whereas the control groups’ consumption increased. In another program (7) with modest design quality evaluation the experimental group even showed a higher frequency of alcohol consumption. In the only long-term study (10) of a family-based program substance consumption was elevated in both study groups (intervention and control group) compared to other population samples, and the risk for developing SUD in adolescence or young adulthood was significantly reduced for males, but elevated for females. (b) An improvement in *coping strategies* was a central part of almost all studied programs, with the exception of one (9/10). Frequently, an improvement was observed. In one study with good design quality (3), only girls showed better coping strategies. (c) *Social behavior* was also frequently assessed and showed significant improvements in all studies, especially for family-based programs, but also otherwise. (d) *Self-worth* enhancement was assessed in four programs with inconsistent findings. One study with good design quality found improvement of self-worth (5), but only for a group that received additional training as mentors (not as mentees). A study with poorer design quality reported increase of self-worth, but the duration of the program was over two years, i.e. untypically long (7/8). Two further high quality studies did not reveal significant effects on self-worth (1/2). (e) *Program-related knowledge* such as facts about alcohol, drugs, addiction, and their effects on families was assessed in five of the studies (1/3/4/6/12) and increased substantially in all cases.

(f) *Unexpected findings / negative effects* also occurred: positive alcohol expectations rose in one study (1) with very good design quality, even though the intended effect was the opposite. In the same study no outcome differences between groups with or without individual trainer component were found. This finding contrasts with another study, also with good design quality, in which positive effects of mentorship were reported (5). Also, high levels of loneliness and isolation were found at pre-test measurement in one study (2) with good design quality, which did not change after the 8-week program. In another study of low quality (6) that featured 11 sessions plus a mentorship component participants did report decreased levels of isolation. In a further study (3; good design quality) there were other unexpected findings such as increased medical complaints and diminished social integration for boys. In one program (5; good design quality), positive effects were also reported for the wait control groups, while this was either not the case or not reported in other programs.

In our review, we frequently find two studies focusing on the same program. Results could be interpreted more definitely if they were repeated in programs implemented several times. However, this is not the case in the studies examined here: In almost all cases, we find additional or follow-up information on only one program implementation (3/4, 7/8, 9/10). Only one program was implemented twice (5/6), but with very different sample sizes and different results, the only repeated result being school performance.

## Discussion

The existing body of research on prevention programs for children in substance-affected families was reviewed, and some key points can be highlighted. While some school-based studies showed many effects, others did not, and while one family program showed very little effect (9/10), the SFP adaptations showed good results (11–13). A fairly homogeneous finding is the substantial increase in program-related knowledge in all studies that included this outcome measure. The weighted ES for knowledge was large with *r*(+) = .55. This demonstrates the effectiveness of psycho-educative program components. Self-worth was rarely enhanced by the programs with the exception of participants in an intervention that had received additional mentor training (5). The fact that only a multi-component group and a very long-term program (SFP) had any effects in this area indicates that self-worth may be an attribute of trait quality, and, therefore, not easy to improve. It is subject to many influences and long-term developments that are deeply rooted in identity issues. This seems to be different for coping strategies which improved frequently. The weighted ES for coping was medium sized with *r*(+) = .34. However, in one study with good design quality (3), only girls showed better coping strategies, an effect that the researchers attribute to the age group of the study (M = 15.5 years) in which girls may be more mature for developmental reasons. Further research on the interplay of age, gender and program effects is needed to explore differential aspects in tapping the coping resources of these children.

The frequent findings on improvement of social behavior are rendered somewhat inconclusive by the fact that no study described baseline social behavior levels. On the other hand, it is made more credible by the fact that social behavior improvements were not only reported by the children themselves, but also by parents and/or teachers. The weighted ES for social behavior was small with *r*(+) = .17. One possible reason for the lack of statistically significant reductions in substance use may be that many of these studies were conducted with children younger than 12 years of age. A majority of them probably were not consuming substances at this point. Longitudinal studies examining the development of own substance use at a later point in children’s development were rarely funded in the past. On the whole, the effect of preventive programs for children of substance-affected families on their own problems with substances remains unclear and merits more longitudinal research.

Over all studies providing appropriate data (see Table 4), and assuming homogeneity in overall outcome, ES would be *r*(+) = .33 or d(+) = .70, meaning that any randomly chosen child in the treatment conditions had a 69% probability to benefit from the prevention program. From this, we conclude that even though some study results seem promising, evidence is still too mixed for definite conclusions on “what worked best”. This is especially true for school-based programs. For instance, unexpected effects occurred such as the elevation of positive alcohol expectancies (1), or the increase of health complaints and decrease of social integration for boys (3). In other studies, negative findings were not mentioned – it is unclear whether they occurred or not. Even though some explanations are offered for negative effects authors generally made no suggestions how to deal with these effects. Future research should attempt to discuss such findings in the light of possible modifications of programming, recruiting or study design. Editors and funders should require reports on adverse events and findings. The success of short intervention programs may rest on the emphasis placed on tailoring programs to the needs of their target group, for instance, to age, gender, or cultural background. Programs that contained components over a longer period of time (i. e. over half a year or longer) produced superior effects compared with shorter interventions. Therefore we tentatively conclude that SMAAP (1) and CHOICES (5/6) were the programs with the best evidenced effects, and that CHOICES is superior to SMAAP because of its multi-component approach and long-term components. Teen-Club (7/8) also showed positive long-term effects, but the intervention was not well studied.

The success of family-based programs, especially SFP, is clearer and points to the value of integrating both parents and children into programs, wherever possible. This is a disadvantage of school-based programs, where parents hardly participated. On the other hand, recruiting substance-involved parents to participate in a program of this kind can be extremely difficult, so that low-threshold interventions remain necessary. Special “success factors“ in a family setting appear to be a focus on the family level, not only the individual level, and a broad integrated approach to many kinds of problem behavior. The program that focused more on parent training was less successful. This is in line with the research on parenting described in the Background section and with the conclusions stated by Kumpfer and Alvarado [47] in their reviews of family-based prevention programs. The weighted ES for family functioning was on a medium level with *r* (+) = .27.

Each of the nine programs except for the community-based one (7/8) was evaluated in at least one very good or at least good quality study. Often large sample sizes were employed and multiple change indices, assessed in different ways, but often with standardized instruments, were employed. This adds credibility to the findings described and synthesized in this article, even though the small study base must be taken into account. The following limitations to the body of research presented here point to conclusions for future research.

*(a) Quantity of studies:* Only 13 studies of 375 titles (3.5%) were included, representing to our knowledge all of the published work in this area conducted over 15 years of research (1994–2009). Almost no published work from other countries besides the United States exists to date. Clearly more programs and research agendas in different cultural and contextual settings are needed for this high-risk target group in the future. *(b)Sample sizes:* These varied considerably. They should be based on thorough power analysis to achieve adequate statistical power and enable subgroup analyses. *(c) Treatment integrity:* Even though most programs had well-trained staff, adherence to the program was often not reported. In some studies research staff also administered the program, which creates a possible bias. Future research should seek to document treatment fidelity in audio- or videotape analysis, not relying on group leader reports alone.

*(d) Measurement of effects:* The most important independent variable, parental substance use, was often not validated rigorously, but assessed solely by a short screening question or child self-report. A multitude of parameters were assessed, in many cases only by self-report of the children, sometimes by teacher or group-leader report, rarely by parental assessment. As often is the case, participating teachers and parents were not blinded to treatment condition, creating a consistent bias in all of the studies. Even though own substance problem prevention seemed the primary goal in most studies this was rarely assessed directly. Instead, it was assumed that certain indicators (e.g., behavioral problems, family relations, psychosocial adjustment) would indirectly contribute to achieving this goal. Future research should aim at clearly defining treatment goals and using validated instruments already utilized in other studies to enhance study comparability. Multiple perspectives, including parent perspective, should be employed to validate children’s self-reports. *(e) Recording adverse events:* Adverse events, i.e. adverse changes in health or side effects during program delivery are hardly reported in the literature mentioned here and in other current literature. This is a serious and important omission [82-84]. Documenting adverse events or their absence can uncover possible harm done by delivering the program. For instance, in the case of children from substance-affected families, their being educated about the parental problems without including parents in the intervention could cause serious conflict and hostility within a vulnerable family.

*(f) Follow-ups:* Most studies relied on pre-post-tests, only some studies conducted follow-ups (1/7/8/9/10), two studies of which were of inferior quality (7/8). These studies showed that effects tended to decrease over time. Nevertheless, it seems likely that in the context of parental substance use and for this age group, program effects may be more long-term, especially regarding own substance consumption status, but also regarding other psychosocial developments. Future research must seek to conduct longitudinal studies to identify possible delay effects as are found in meta-analytical family therapy research [85,86], or their absence. *(g) Component analysis:* By employing wait-control lists no program effects were compared to effects of unspecific or different interventions. Therefore almost no study focused on which component was important for producing desired effects. Only study (5) compared effects of different components and found that participants receiving two instead of one component showed better results. A next generation of research must identify success factors in programs, i.e. effective program ingredients, to enhance efficiency and effectiveness of preventive interventions for children of substance abusing parents.

## Conclusions

All forms of intervention, i.e., school-based, community-based, and family-based interventions, showed valuable results, but these are found in a very small number of program evaluation studies. Thus, while there is evidence for programs’ effectiveness in reducing high-risk children’s problems and improving positive behaviors, coping skills, and feelings, it remains preliminary. It is up to future work to broaden the body of research on programs for children from substance-affected families. Next to testing new approaches to prevention, using less expensive implementation methods such as web and DVD-based programs for wider dissemination in different contexts and with different age groups, more work is needed that explores different age groups and settings as well as cultural particularities in different countries. Program developers can draw on best practices identified in the broader area of family-based prevention programs [87]. For the public health system, the mixed results found here also call for investing more in carefully planned program evaluations to better allocate funding to effective programs. Also, more emphasis should be placed on program implementation after the evaluation is finished. This would enable re-testing program effects in more naturalistic settings. Funders should especially consider supporting efforts aimed at identifying long-term effects of prevention programs for children from substance-affected families. The fulfillment of the ultimate program goal, i.e., to reduce intergenerational transmission of SUD and other mental health problems, cannot be determined by any other means than by longitudinal research.

## Competing interests

Prof. Karol Kumpfer is a co-officer in Lutra Group, Inc. that disseminates the SFP curriculum, delivers the trainings, and conducts the evaluations. We declare that no further conflicts of interest exist. The authors declare that they have no competing interests.

## Authors’ contributions

SB, KKu, KKr planned the design and drafted the manuscript. P-MS contributed methods consulting and critical revision. KKr, IS-B interpreted and analyzed data, ISB, SR, DM, EP collected data and researched articles. MK, RT were involved in all parts of the review as general supervisors of the research group. All authors read and approved the final manuscript.

## Authors’ information

Most authors of this paper work at the two partner institutes currently conducting a large national trial of a community-based intervention for children from substance-affected families in Germany (http://www.projekt-trampolin.de). For more information on our research, see http://www.dzskj.de, http://www.disup.de.
